# Oropharyngeal microbiome profiled at admission is predictive of the need for respiratory support among COVID-19 patients

**DOI:** 10.3389/fmicb.2022.1009440

**Published:** 2022-09-30

**Authors:** Evan S. Bradley, Abigail L. Zeamer, Vanni Bucci, Lindsey Cincotta, Marie-Claire Salive, Protiva Dutta, Shafik Mutaawe, Otuwe Anya, Christopher Tocci, Ann Moormann, Doyle V. Ward, Beth A. McCormick, John P. Haran

**Affiliations:** ^1^Department of Emergency Medicine, UMass Memorial Medical Center, Worcester, MA, United States; ^2^Program in Microbiome Dynamics, University of Massachusetts Medical School, Worcester, MA, United States; ^3^Department of Microbiology and Physiologic Systems, University of Massachusetts Medical School, Worcester, MA, United States; ^4^Department of Biology and Biotechnology, Worcester Polytechnique Institute, Worcester, MA, United States; ^5^Department of Medicine, University of Massachusetts Medical School, Worcester, MA, United States

**Keywords:** oropharyngeal microbiome, COVID-19, SARS-CoV-2, random forest classification, commensal organisms, *Prevotella*, LPS biosynthesis

## Abstract

The oropharyngeal microbiome, the collective genomes of the community of microorganisms that colonizes the upper respiratory tract, is thought to influence the clinical course of infection by respiratory viruses, including Severe Acute Respiratory Syndrome Coronavirus 2 (SARS-CoV-2), the causative agent of Coronavirus Infectious Disease 2019 (COVID-19). In this study, we examined the oropharyngeal microbiome of suspected COVID-19 patients presenting to the Emergency Department and an inpatient COVID-19 unit with symptoms of acute COVID-19. Of 115 initially enrolled patients, 50 had positive molecular testing for COVID-19+ and had symptom duration of 14 days or less. These patients were analyzed further as progression of disease could most likely be attributed to acute COVID-19 and less likely a secondary process. Of these, 38 (76%) went on to require some form of supplemental oxygen support. To identify functional patterns associated with respiratory illness requiring respiratory support, we applied an interpretable random forest classification machine learning pipeline to shotgun metagenomic sequencing data and select clinical covariates. When combined with clinical factors, both species and metabolic pathways abundance-based models were found to be highly predictive of the need for respiratory support (F1-score 0.857 for microbes and 0.821 for functional pathways). To determine biologically meaningful and highly predictive signals in the microbiome, we applied the Stable and Interpretable RUle Set to the output of the models. This analysis revealed that low abundance of two commensal organisms, *Prevotella salivae* or *Veillonella infantium* (< 4.2 and 1.7% respectively), and a low abundance of a pathway associated with LPS biosynthesis (< 0.1%) were highly predictive of developing the need for acute respiratory support (82 and 91.4% respectively). These findings suggest that the composition of the oropharyngeal microbiome in COVID-19 patients may play a role in determining who will suffer from severe disease manifestations.

## Introduction

COVID-19, caused by the severe acute respiratory syndrome coronavirus 2 (SARS-CoV-2), has sickened an estimated 605 million people and caused 6.5 million deaths, likely an undercount, since the start of the pandemic, of those nearly 95 million cases and in excess of one million deaths in have been in the United States alone (Dong et al., 2020). A distinct feature of this disease that has presented unique challenges for the healthcare community is the variability of disease symptoms and clinical course in patients. Some individuals develop severe disease and death rapidly while others present with only mild or no symptoms (Bai et al., 2020). Additionally, some patients go on to develop persistent symptoms that last beyond 12 weeks, known as post-acute or long COVID (Nalbandian et al., 2021) with or without severe illness during initial infection (Crook et al., 2021). While certain clinical co-factors, such as age, Body Mass Index (BMI), and medical comorbidities, in combination with initial vital signs, and clinical laboratory testing, are currently being used to predict clinical decompensation and the need for ICU level of care (Haimovich et al., 2020; Gallo Marin et al., 2021), these clinical variables do not shed light on the exact pathophysiologic mechanisms leading to acute respiratory illness or severe disease outcomes. There are likely other individual factors that determine how a patient responds to SARS-CoV-2 and which may play a role in determining the various disease manifestations of COVID-19 (Beck and Aksentijevich, 2020).

Evidence is emerging that the microbiome can influence clinical disease in COVID-19. Dysbiosis in both the gut and oral microbiomes have been associated with disease symptoms or severity (Bao et al., 2020; Ren et al., 2021; Yeoh et al., 2021; Zuo et al., 2021). The oropharyngeal microbiome has been suggested to impact symptoms in COVID-19 and influence inflammation within the oral cavity (Soffritti et al., 2021). Specific dysbiotic oral microbiota have been associated with biomarkers of inflammation in COVID-19 patients (Iebba et al., 2021, Cui et al., 2022). Adding to this evidence, our group recently discovered associations between the oral microbiome and long COVID. Specifically, higher abundances of certain pro-inflammatory and lipopolysaccharide producing microbiota and lower abundances of genes for anti-inflammatory metabolic pathways were linked to developing long COVID, likely through promotion of a systemic inflammatory state in certain patients (Haran et al., 2021a). Complex interactions between microbiome-host axes (e.g., oral – lung – aspiration axis, oral/gut – systemic axis, gut – brain axis, etc.) may help explain some of the variability in host immune responses in COVID-19.

The oropharyngeal and nasopharyngeal microbiomes, the collective genomes of microorganisms that colonize the human upper airway, have been hypothesized to influence host immune responses to respiratory viral and bacterial infections (Man et al., 2017). Viral co-infection in the upper airway and lungs may promote bacterial pathogens by mechanisms such as liberating nutrients, exposing adhesion molecules, induction or enhancement of bacterial virulence gene expression, and priming of host environment and immune responses to become more susceptible to infection (Avadhanula et al., 2006; Mccullers, 2014; Li et al., 2015; Bakaletz, 2017), leading to more severe disease and secondary or polymicrobial bacterial infection. Conversely, commensal bacterial species of the nasopharynx can modulate the immune response to influenza virus infection in a potentially protective way (Abt et al., 2012; Short et al., 2014). Upon initial infection, SARS-CoV-2, would most likely first encounter the nasopharyngeal and oropharyngeal microbiota, where similar microbiome-host immune system interactions may influence the course of disease. Just as specific signatures of the oral microbiome were predictive of a post-acute COVID disease manifestation in our recent work and other aspects of COVID-19 associated sequelae in work by others, we predict that specific patterns in the oropharyngeal microbiome may also be associated with acute phase disease outcomes, specifically the development of severe acute respiratory illness.

Here, we hypothesize that the makeup of the oropharyngeal microbiome, including the presence and relative abundance of microbial species as well as metabolic gene content, collected at admission may be predictive of the clinical trajectory of acute respiratory illness in COVID-19, specifically in terms of the need for receiving respiratory support (ranging from supplemental oxygen by nasal cannula to intubation). To test this hypothesis, we investigated the oropharyngeal microbiome of individuals presenting with symptoms suggestive of COVID-19 and positive molecular testing for SARS-CoV-2. We used machine learning-based modeling to examine associations between the microbial residents of the oral cavity and a manifestation of severe COVID-19, specifically, the need for respiratory support. To extract easily interpretable predictive rules from the machine learning model, we then implemented a rule-based classification algorithm. These rules utilize the measured abundance of specific bacterial species and metabolic pathways identified by our machine learning models to give insight into how specific features of the oropharyngeal microbiome impact the clinical course of COVID-19, specifically with respect to why some patients need respiratory support during initial SARS-CoV-2 infection. Our findings further strengthen the links between microbiomes of the upper airways and COVID-19 disease manifestations.

## Materials and methods

### Enrollment

Patients presenting with COVID-19 symptoms at the UMass Memorial Medical Center Emergency Department or while admitted to UMass Memorial COVID-19 treatment units were approached for enrollment in the study. Some individuals had known COVID-19 status when approached on inpatient COVID-19 wards, but the majority were approached in the Emergency Department prior to receiving results of molecular tests for SARS-CoV-2. Enrollment and sample collection took place April 2020 through March 2021, before vaccines were widely available, and no subjects had been vaccinated against COVID-19. Enrolled patients were followed prospectively through the Electronic Medical Record (EMR). We collected information on disease outcomes of COVID-19 for their initial visit including need for respiratory support, the results of all laboratory testing, and mortality *via* the EMR.

### Classification of samples

Oropharyngeal samples were classified as being collected from a patient with acute COVID-19 (COVID+) if they had a documented positive rtPCR testing for SARS-CoV-2 and self-reported symptoms for 14 days or less. The need for respiratory support was classified as positive if the patient required any intervention to support breathing. This included supplemental oxygen *via* nasal cannula or face mask, non-invasive positive pressure ventilation, or intubation. If a patient had a Do Not Intubate (DNI) order but died of COVID-19 symptoms, we considered that patient has having respiratory failure severe enough to require intubation and classified the sample as being from a patient who was intubated. Patients were considered as having in-hospital mortality from COVID-19 if this was listed as a cause of death on hospital death records.

### Sample collection and processing

Oropharyngeal samples were collected using OMNIgene•ORAL collection kits (OMR-120, DNA Genotek). Briefly, the posterior oropharynx was swabbed for 30 s and collected as per manufacturer protocol. Samples were heated at 65–70°C for 1 h (Rabenau et al., 2005) to ensure SARS-CoV-2 inactivation and then stored frozen at −20°C. Upon thawing for nucleic acid extraction, samples were treated with 5ul Proteinase K (P8107S, New England Biolabs) for 2 h at 50°C, then extracted manually by laboratory staff using ZymoBIOMICS DNA/RNA Miniprep Kits (R2002, Zymo Research) as per manufacture protocol in a dedicated lab space within a biosafety cabinet separate from library preparation areas. DNA sequencing libraries were constructed using the Nextera XT DNA Library Prep Kit (FC-131-1,096, Illumina) and sequenced on a NextSeq 500 Sequencing System as 2 × 150 nucleotide paired-end reads.

### Sequence processing and analysis

Shotgun metagenomic reads were first trimmed and quality filtered to remove sequencing adapters and host contamination using Trimmomatic (Bolger et al., 2014) and Bowtie2 (Langmead and Salzberg, 2012), respectively, as part of the KneadData pipeline version 0.7.2.^1^ As in our previous work (Haran et al., 2018, 2019b), reads were then profiled for microbial taxonomic abundances and metabolic pathways using Metaphlan3 and HUMAnN3, respectively (Truong et al., 2015; Beghini et al., 2021). Samples with very low bacterial diversity (< 4 individual species detected) were discarded as these swabs contained very few bacterial reads.

### Microbiome-clinical factors modeling

To determine the association between microbial species abundance and COVID-19 diagnosis, we performed a non-parametric Wilcoxon Rank Sum test for species with at least 5% prevalence and a minimal average relative abundance of 0.01% across all samples (*n* = 115; 74 COVID-19+ and 41 COVID-19–) with the Bonferroni correction for multiple comparisons.

To identify oropharyngeal microbes and clinical covariates that are predictive of needing respiratory support in acute COVID-19+ patients and compare their relative contributions, we developed and ran a random forest classification (RFC)-based pipeline in R. For each subset of data, the pipeline was run six times from six different random seeds, and statistics for the model’s classification performance and variables contribution to class discrimination were calculated for each seed. The first step of the pipeline is a leave-one-out cross-validation split of the data. Leave-one-out cross-validation is a model validation scheme in which a data set of *n* observations is split into a training and test set where all but one observation is part of the training set; thus one observation is left out. The training set, with *n-1* observations, is used to build and train a model. The resulting model is then used to predict the left-out test observations and calculate performance statistics. This process is repeated *n* times.

In our pipeline, the training set resulting from each leave-one-out cross-validation fold was used for the following steps of the pipeline. Feature selection using Boruta (Kursa and Rudnicki, 2010) was run in a leave-one-out cross-validation scheme to select a subset of variables that are discriminatory. The Boruta-selected variables were then used to train a RFC model, using the ranger package (Wright and Ziegler, 2017). The resulting RFC model was then used to predict the left-out sample and calculate permutated variable importance. Thus, the performance of our model is calculated based on the aggregated predictions of left-out data. Using the features of the top performing data subsets and models, we applied Stable and Interpretable RUle Set (SIRUS; Bénard et al., 2021) to extract a list of rules informative of and highly predictive of respiratory support. Rules are displayed on the top of violin plots depicting the feature described by each rule. Plots were generated in R using the ggplot2 package (Wickham, 2016) and color palettes from the calecopal package.^2^ Conceptual figures were generated with the aid of BioRender.com.

## Results

### Patients requiring respiratory support were similar to those who did not

**Figure 1:**
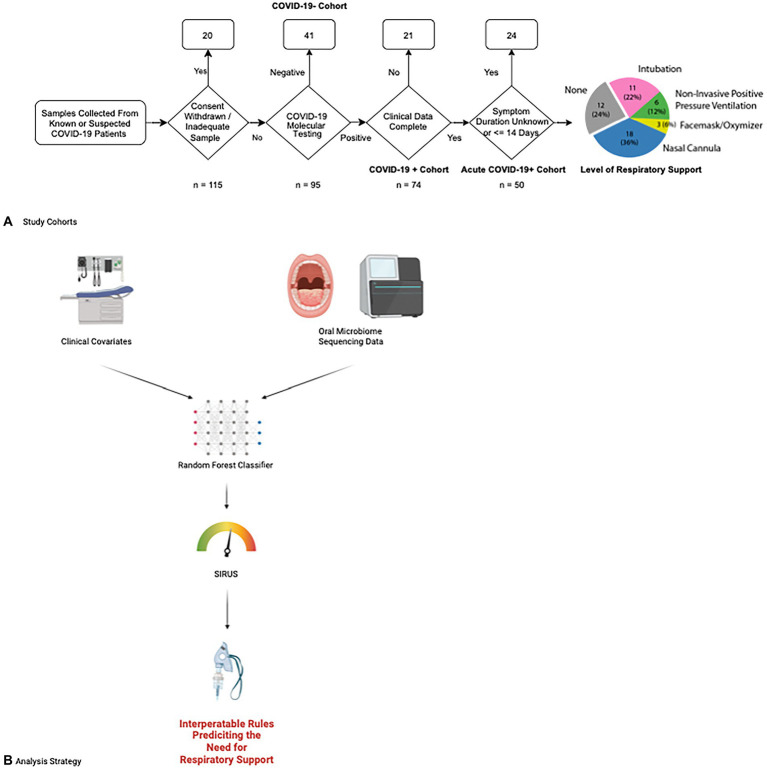
Study enrollment and data analysis flowcharts. **(A)** Patients at UMass Medical center were enrolled for our study according to the following flow chart. Fifty patients with acute COVID-19 were ultimately selected for our study cohort and followed for a clinical outcome of whether they needed respiratory support and what level of respiratory support was required, ranging from supplemental oxygen *via* simple nasal cannula escalating through intubation and mechanical ventilation. The number of patients requiring each level of respiratory support is shown in the final chart on the right. **(B)** Data from clinical covariates and microbiome sequencing results are combined in a random forest classifier to determine features predicting the need for respiratory support. We then applied the Stable and Interpretable RUle Set (SIRUS) to these results to generate easily interpretable rules predicting which clinical covariates and microbiome features are predictive of the need for respiratory support.

We prospectively collected oral microbiome samples from 115 patients, 50 of whom ultimately tested positive for COVID-19 and had been experiencing symptoms for up to 14 days prior to hospital presentation (Figure 1). Our overall COVID-19+ and COVID-19− cohorts were overall similar with the exception of the COVID-19+ cohort having a higher portion of former or active smokers (Supplementary Table S1) Examining our acute COVID-19+ cohort, 38 (76%) required some form of respiratory support. Except for Body Mass Index (BMI; *p* < 0.05), our cohort of COVID-19+ patients had similar characteristics between those requiring respiratory support and those who did not (Table 1). The overall mean age of the final cohort was 68 (SD 15.24); 50% were female and most patients identified as Hispanic or Latino (76%) and white (64%). Within the acute COVID+ cohort (Figure 1), 12 (24%) patients never required any respiratory support, 18 (36%) were treated with supplemental oxygen *via* nasal cannula, 3 (6%) were treated with supplemental oxygen *via* facemask, 6 patients were treated with positive pressure ventilation (12%), and 11 (22%) were intubated. There were 2 patients who died of COVID-19 but had Do Not Intubate (DNI) orders; accordingly, they were considered as having respiratory failure severe enough to require intubation. Broad measures of microbiome diversity in our samples (Shannon index, Simpson Index, inverse Shannon index) were similar between groups (Table 1).

**Table 1:** Study population characteristics.

		Respiratory support	
Characteristic	Overall, *n* = 50^1^	no, *n* = 12^1^	yes, *n* = 38^1^	*p*-value^2^
BMI	29.12 (7.01)	23.83 (5.11)	30.79 (6.74)	0.003
Age	68.00 (15.24)	60.83 (19.52)	70.26 (13.12)	0.15
Male (%)	25/50 (50)	5/12 (42)	20/38 (53)	0.5
*Ethnicity*				
Caucasian (%)	32/50 (64)	5/12 (42)	27/38 (71)	0.089
Black (%)	5/50 (10)	2/12 (17)	3/38 (7.9)	0.6
Asian (%)	2/50 (4.0)	2/12 (17)	0/38 (0)	0.054
Other (%)	11/50 (22)	3/12 (25)	8/38 (21)	>0.9
Hispanic or Latino (%)	38/50 (76)	7/12 (58)	31/38 (82)	0.13
CCI	4.50 (2.58)	3.75 (3.05)	4.74 (2.41)	0.2
Hypertension (%)	33/50 (66)	7/12 (58)	26/38 (68)	0.7
Diabetes (%)	18/50 (36)	5/12 (42)	13/38 (34)	0.7
Asthma (%)	8/50 (16)	1/12 (8.3)	7/38 (18)	0.7
COPD (%)	10/50 (20)	2/12 (17)	8/38 (21)	>0.9
OSA (%)	3/50 (6.0)	0/12 (0)	3/38 (7.9)	>0.9
Smoker, current (%)	1/50 (2.0)	1/12 (8.3)	0/38 (0)	0.2
Smoker, former (%)	21/50 (42)	3/12 (25)	18/38 (47)	0.2
COVID fatality (%)	8/50 (16)	0/12 (0)	8/38 (21)	0.2
*Diversity measures*				
Shannon	2.25 (0.62)	2.50 (0.35)	2.17 (0.66)	0.2
Simpson	0.80 (0.13)	0.86 (0.04)	0.78 (0.15)	0.3
Inverse Simpson	7.04 (3.69)	7.70 (2.39)	6.83 (4.02)	0.3

1 Mean (SD); *n*/*N* (%).

2 Wilcoxon Rank Sum test; Fisher’s exact test; Pearson’s Chi-squared test.

CCI, Charlson Comorbidity Index; COPD, chronic obstructive pulmonary disease; OSA, obstructive sleep apnea.

### No reliable differences were detected between COVID-19− and COVID-19+ patients

Shotgun metagenomic sequencing was performed on all collected oropharyngeal microbiome samples. Human DNA sequences were filtered out prior to downstream analysis (mean metagenomic reads/sample 4,395,936 ± 457606.9). Resulting sequences were profiled for microbial abundances and metabolic pathways. Samples with very low diversity (< 4 detected microbial species) were discarded as these contained mostly contaminating human DNA. Reads coming from host contamination versus microbial species was not significantly different between COVID-19+ and COVID-19− cohorts (Mann–Whitney U-test *p* = 0.548, and Supplementary Figure S1). The portion of host contamination reads in samples from COVID-19+ participants requiring respiratory support versus those who did not was also not significantly different (Mann–Whitney U-test 0.2382, Supplementary Figure S1). We directly compared abundances of microbiome features between COVID-19+ and COVID-19− patients utilizing the Wilcoxon Rank Sum test. When using the Bonferroni correction for multiple comparisons, there was no differential abundance of microbial species or metabolic pathways that were significantly different between COVID-19+ and COVID-19− patients (Supplementary Tables S1, S2).

### Machine learning-based modeling is effective at extracting microbiome features associated with a clinical outcome (need for respiratory support) from complex multimodal data

As traditional statistical methods often fail to provide meaningful insight into microbiome data due to the heterogeneity, sparsity and dimensionality issues common to microbiome data sets, we next used machine learning (ML) modeling for analysis. ML is apt for overcoming the mentioned challenges and enables the integration of complex, highly variable microbiome data and clinical variables (Cammarota et al., 2020). RFC ML models, especially suit microbiome data because they enable the use of non-normally distributed data (such as species relative abundance) and diverse sets of variables (Shannon’s alpha diversity index, and numerical and categorical clinical covariates) as features in the same model, thus allowing for prediction of clinical responses from complex multimodal data (Haran et al., 2019a; Wipperman et al., 2021). We have previously demonstrated that RFC models can discover robust correlations between the microbiome and clinical outcomes in various diseases (Haran et al., 2019a, 2021a,b; Wipperman et al., 2021; Bradley et al., 2022). For this study, we are also extending our ML pipeline with the Stable and Interpretable RUle Set (SIRUS) algorithm (Bénard et al., 2021), which allows for simultaneous extraction of highly predictive, human interpretable, and clinically relevant rules on how microbiome features associate with clinical outcomes. For example, the algorithm found as the most prevalent rule that “if *Prevotella salivae* abundance is < 4.3% the probability of needing respiratory support is 82.2%.”

**Figure 2:**
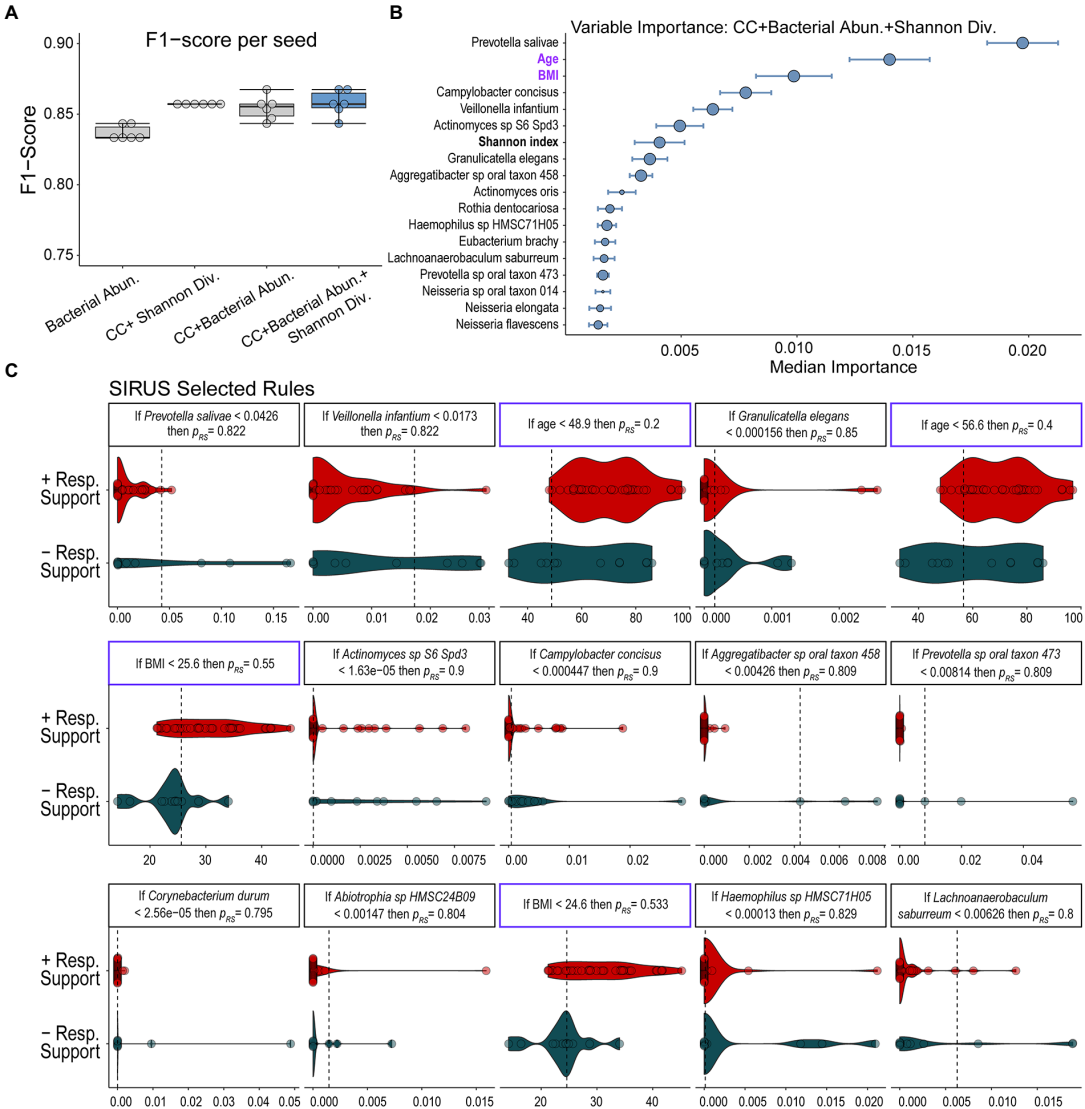
Results of random forest classification model. **(A)** F1-scores of RFC models including clinical covariates (CC), individual microbial abundances, and the combination of bacterial abundances, alpha diversity, and clinical covariates show that all models perform well with models including all multimodal data performing slightly better. **(B)** Median ranked importance of features from the final model (blue boxplot) trained on all data including microbiome features, alpha diversity, and clinical data (median importance ± median absolute deviation) are shown. The size of the circle represents how often each feature was selected. The relative abundance of *Prevotella salivae* is the top predictor with the relative abundance of *Campylobacter concisus*, *V. infantium* and *Actinomycetes* sp. S6-Spd3 and the Shannon diversity index also showing significant contributions. Significantly contributing clinical covariates were age and BMI. Features which do not contribute to the predictive model are not shown. **(C)** The relative abundance of the microbiome features or values of clinical variables determined to be important in predicting the need for respiratory support by our RFC model are displayed along with discriminative rules based on the probability of requiring respiratory support (p_res_).

Using RFC, we sought to define the microbiome’s role in predicting the need for respiratory support in our COVID-19+ cohort. To demonstrate the utility of combining multimodal inputs, we trained our RFC model on (1) patient features alone, (2) microbiome data alone, and (3) microbiome plus clinical data and computed F1-scores from leave-one-out cross-validated data across multiple random seeds (see materials and methods). F1-score is the harmonic mean between precision and recall, which accounts for both prediction errors and the specific type of prediction error. We have chosen the F1-score as our main model evaluation metric because the F1-score accounts for imbalanced data and extremes in either recall or precision while maximizing both. Thus, this metric is best suited for cases where both false positives and false negatives are undesirable. A model trained with host specific features, which we define as both sample-level Shannon’s alpha diversity index and clinical covariates, including age, BMI, ethnicity, and selected medical comorbidities available at admission (Table 1), performed well with a mean F1-score of 0.857 ± 0.000 (Figure 2A). Note that the combination of these clinical covariates has already been determined by other studies to be predictive of decompensation in COVID-19 (Gallo Marin et al., 2021) and thus help validate our modeling approach. A model trained only on measured microbial abundances performed comparably with a mean F1-score of 0.837 ± 0.005 (Figure 2A). A model including clinical covariates, select medical comorbidities, measured microbial abundances, and sample-level Shannon’s alpha diversity index led to a similar predictive performance as measured by a mean F1-score of 0.858 ± 0.009 (Figure 2A). These F1-scores indicate similar performance of clinical and microbial variables. Additional model statistics are included in Supplementary Table S3. Based on the performance of the model that combined microbiome features, all clinical covariates, and sample-level Shannon’s alpha diversity index, we continued our analysis on results from this trained model to examine how features selected by the model associated with the need for respiratory support in acute COVID-19 in our cohort.

### Relative abundance of certain commensal species is associated with developing illness requiring respiratory support in COVID-19

We further analyzed the results of our RFC model combining microbial species abundance data with patient clinical covariates and sample-level Shannon’s alpha diversity index to determine which microbial populations contributed to predicting the need for respiratory support and how they compared to clinical variables. Based on the median calculated permutated variable importance, a ranking of which features contributed most to the prediction (Wright and Ziegler, 2017), the relative abundance of *P. salivae* is the most important predictor needed to accurately classify an individual for respiratory support outcomes (Figure 2B). Notably, this organism is ranked higher than both patient age and BMI (Figure 2B), which are two clinical factors known to associate with severe COVID-19 (Gallo Marin et al., 2021), and the only two clinical factors determined by the model as predictive. To determine a set of logical rules that separate individuals according to their future need for respiratory support using the microbiome and clinical covariates, we passed the inferred RFC model results to the SIRUS algorithm (Bénard et al., 2021; Figure 1B). SIRUS identified 15 highly predictive logical rules of the form “If the abundance of feature X is greater than Y, then the probability of needing respiratory support is Z” (Figure 2C). SIRUS found that low abundances of *P. salivae* or *Veillonella infantium* (< 4.2 and 1.7% of the microbiota respectively), two microbes found at significant levels, is associated with an 82% probability of needing respiratory support. Both rules were found to be the most selected across the different cross-validation folds and were more generalizable across individuals compared to rules that account for clinical factors/demographics including BMI and age (Figure 2C). We also found that relative abundances of *Actinomyces* and *Campylobacter concisus* contributed moderately to the prediction.

### Profiling of metabolic pathways refines the role of commensal microbiota in prediction of developing acute respiratory illness in COVID-19

While our above modeling provided robust predictions based on certain microbial species, analysis of species abundance alone provides a limited understanding of the exact functional roles of associated species in terms of biologic mechanism with respect to clinical outcomes. To profile the microbiomes functionally and discover possible relationships between microbial metabolic pathways and clinical outcomes, we repeated the RFC and SIRUS modeling pipeline on clinical covariates and the abundance of metabolic pathways, as profiled using HUMAnN3 (Franzosa et al., 2018). Pathway abundance is computed once at the community- and species-level using community- and species-level gene abundances along with established knowledge about the structure of the metabolic pathway. Specifically, each pathway’s abundance in a community is calculated as the sum of the abundances of that pathway’s component reactions. Importantly, pathway abundance is proportional to the number of complete copies of the pathway in the community.

**Figure 3:**
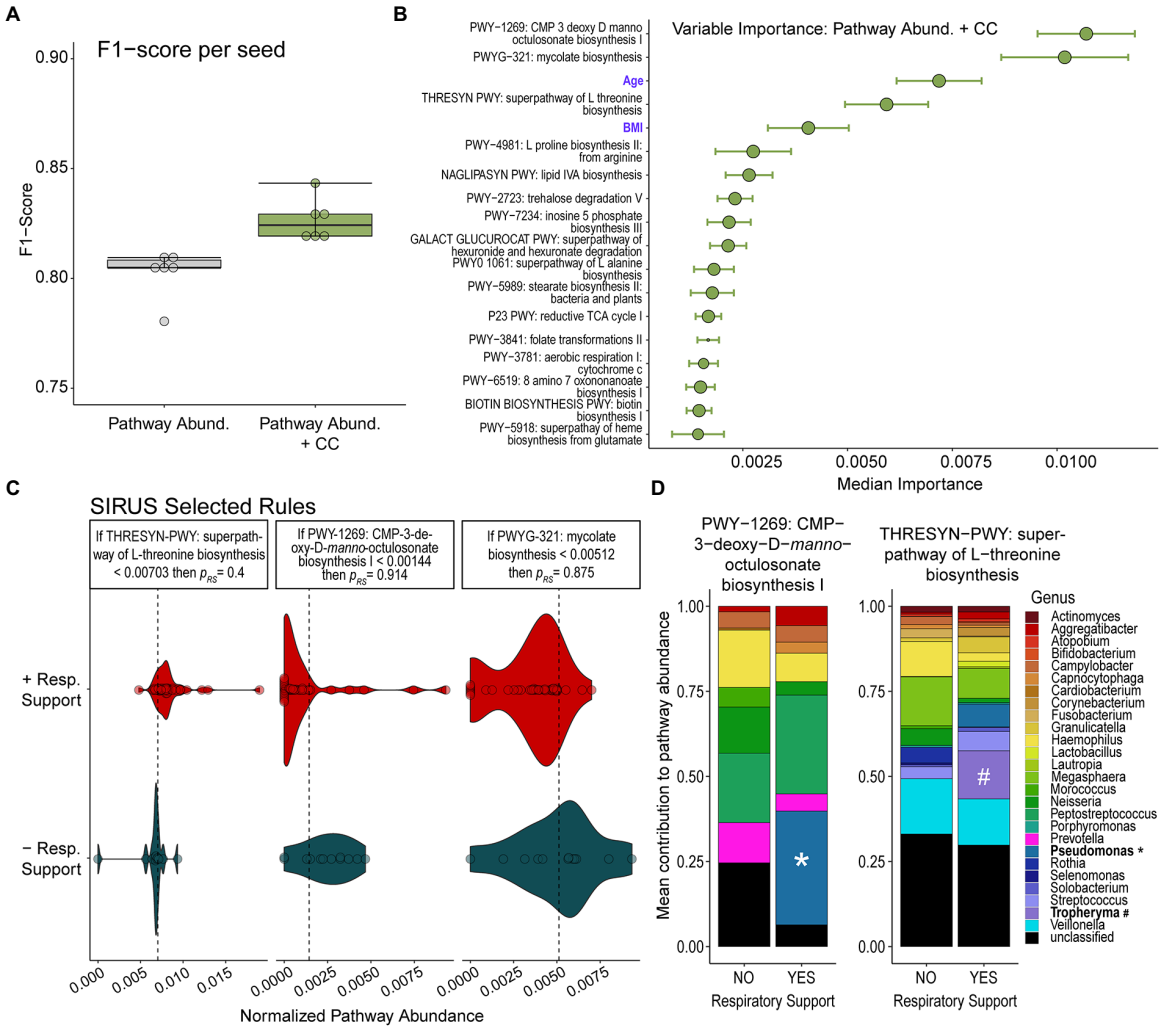
Random Forest Classification Using Metabolic Pathways. **(A)** F1-scores of RFC models built on relative abundance of detected metabolic pathways alone or in combination with clinical covariates (CC) show that models combining both data modalities perform slightly better. **(B)** Median relative importance of variables in predicating the need for respiratory support from the model trained on relative pathway abundances and clinical covariates (median importance ± median absolute deviation) are shown. The size of the circle represents how often each feature was selected. Features which do not contribute to the predictive model are not shown. **(C)** The relative abundance of top metabolic pathways or values of clinical features determined to be important in predicting need for respiratory support by our RFC model are displayed along with discriminative rules based on the probability of requiring respiratory support (p_res_). **(D)** Contributions of detected bacterial genera to pathway abundance of CMP-3-deoxy-D-manno-octusonate biosynthesis and L-threonine biosynthesis in patients who did or did not go on to require respiratory support are shown. The detection of *Pseudomonas* contributing to the abundance of the CMP-3-deoxy-D-manno-octusonate pathway and the detection of *Tropheryma* contributing to the abundance of the L-threonine biosynthetic pathway are notable and highlighted.

The relative abundance of specific microbial metabolic pathways was also highly predicative of respiratory illness leading to the need for respiratory support (mean F1-score 0.804 ± 0.009). Adding clinical covariates available at admission to the model resulted in only a slightly higher F1-score of 0.821 ± 0.004 (Figure 3A). Additional model statistics are included in Supplementary Table S4.

The metabolic pathways most important in predicting the need for respiratory support by our modeling were LPS biosynthesis (CMP-3-D-*manno*-octulosonate biosynthesis), mycolate biosynthesis, and L-threonine biosynthetic pathways (Figure 3B). After running SIRUS, we found that a very low abundance of LPS biosynthesis (CMP-3-D-*manno*-octulosonate biosynthesis) was highly predictive of the probability of needing respiratory support (*p* = 91.4%). Similarly, a very low abundance of mycolate biosynthesis also predicted needing respiratory support with a probability of 87.5%. Higher abundances of pathways encoding L-Threonine biosynthesis was associated with the need for respiratory support, although to a lesser degree than low LPS or mycolic acid (*p* = 60%).

We examined the contribution of bacterial genera to the top three predictive pathways. We observed that in patients who developed the need for respiratory support, more of the CMP-3-deoxy-D-*manno*-octulosonate pathway originated from *Pseudomonas* rather than *Prevotella* (Figure 3C). All the reads assigned to the mycolate biosynthesis pathway (second most predictive) were derived from unclassified microbes. When examining the L-threonine biosynthetic pathway (third most predictive), we observed that *Veillonella* contributed a large fraction and that this fraction was lower in individuals needing respiratory support. Additionally, a significant portion of this pathway was found to originate from *Tropheryma* and *Pseudomonas* which are not represented among patients not requiring supplemental oxygen support.

## Discussion

Here we show that the severity of respiratory illness in acute COVID-19 is associated with certain features of the oropharyngeal microbiome. Specifically, the relative abundances of several Gram-negative and *Actinomyces* species and metabolic pathways associated with LPS, mycolic acid, and amino acid biosynthesis are predictive of whether patients will go on to require respiratory support. Using an interpretable machine learning model to examine the importance of specific factors, we found that decreased abundances of *P. salivae*, *C. concisus, V. infantium* and an *Actinomyces* species were highly associated with the need for respiratory support. More importantly, a lack of *P. salivae* and *V. infantium* was found as the most generalizable signal differentiating individuals needing respiratory support versus not. This signal was found to be more robust in outcome classification compared to any signal derived by the model when considering other clinical factors known to impact the need for respiratory support in acute COVID-19. This suggests that the presence of these organisms may be protective against respiratory failure in patients suffering from acute COVID-19. When examining the contributions of specific microbial metabolic pathways, a higher abundance of genes encoding metabolic pathways for LPS biosynthesis (CMP-3-deoxy-D-manno-octulosonate biosynthesis), mycolate biosynthesis, and a lower abundance of genes associated with L-threonine biosynthesis were found to be protective against severe respiratory manifestations of COVID-19 requiring respiratory support. By combining analysis of microbial abundances with metabolic pathways, we can gain deeper insight into microbiome “profiles” which may be predictive of certain clinical outcomes.

L-threonine is an essential amino acid and an increased abundance in bacterial pathways associated with its synthesis was the third most important metabolic pathway predictor of needing respiratory support in our cohort. L-threonine’s association with inflammation has been studied in the context of animal models of colitis, where exogenous supplementation to inflamed tissues prolonged inflammation (Gaifem et al., 2018; Xie et al., 2021). When we examined the contribution of different genera to the L-threonine biosynthetic pathway abundance, we observed the genera *Tropheryma*, a potential respiratory pathogen (Bousbia et al., 2010) and *Pseudomonas,* a known opportunistic respiratory pathogen, represented among individuals who required respiratory support, but not those who did not require respiratory support. While our evidence here is only suggestive, it would be consistent with a hypothesis which favors potential pathogens showing greater contributions to disease through key metabolic pathways, even if their relative abundances are not significantly different.

A lower abundance of mycolic acid biosynthesis genes was the second most important predictor of needing respiratory support in acute COVID-19 by our modeling of metabolic pathways. Mycolic acid has been reported to have immune modulatory activity, including suppression of allergic inflammatory responses (Obihara et al., 2005; Korf et al., 2006; Kim et al., 2014). While our metabolic pathways analysis did not identify specific genera responsible for the mycolic acid production, our modeling results using microbial abundances did find that a lower abundance of several *Actinomyces* were predictive of the need for respiratory support. *Actinomyces* is the only genera found to effect COVID-19 in this study that is hypothesized to be capable of producing mycolic acid (Collins et al., 1985). *Actinomyces* are slow-growing, facultatively anaerobic, Gram-positive organisms and likely a component of a healthy oropharyngeal microbiota (Bowden, 1996; Kononen and Wade, 2015). In a study of the oropharyngeal microbiome among healthy adults, higher *Actinomyces* abundance was associated with decreased systemic inflammation (Demmer et al., 2017). An anti-inflammatory effect provided by mycolic acid potentially produced by *Actinomyces* would support a hypothesis favoring commensal organisms offering protective benefits against pathogens and disease sequelae.

There is likely an interplay between the balance of pro-inflammatory, pro-disease effects versus protective effects by the resident microbiota. Our seemingly contradictory findings with *P. salivae* and LPS may illustrate this point. *Prevotella* are Gram-negative anaerobic organisms and common oropharyngeal colonizers that have been implicated in periodontal disease as well as COVID-19 disease severity (Yang et al., 2012; Khan and Khan, 2020). Decreased *P. salivae* abundance was the strongest and most general predictor of the need for respiratory support in our analysis using microbial abundances. While *Prevotella* has generally been implicated in chronic inflammation (Larsen, 2017), it is also part of normal, healthy oral and lung microbial communities (Bao et al., 2020; Khatiwada and Subedi, 2020). *P. salivae* has been shown in animal models to stimulate less inflammatory cytokine production and lead to less neutrophil chemotaxis than the Gram-negative respiratory pathogens *Moraxella catarrhalis* and *Haemophilus influenzae* (Larsen et al., 2015). It is hypothesized that a penta-acylated LPS produced by *Prevotella* stimulates less innate-immune receptor activation than hexa-acylated LPS produced by Gram-negative respiratory pathogens (*H. influenzae, Pseudomonas aeruginosa*; Brix et al., 2015) and *Escherichia coli* (Larsen, 2017). *Veillonella* is another Gram-negative bacterial genera commonly found in the oropharynx associated with periodontal disease (Delwiche et al., 1985) that also is predicted to produce penta-acylated LPS (Brix et al., 2015). The production of hypo-acylated LPS that generates less inflammation may represent an adaptation that allows *Prevotella* and *Veillonella* species to colonize the upper airway without causing disease.

A recent study found the presence of *P. salivae* and *V. infantium* in the oral cavity to be predictive of COVID-19 among a discriminating set of six species as assessed by 16S sequencing and network systems modeling (Iebba et al., 2021). Patients in this particular study were hospitalized and received supplemental oxygen therapy, but not intubation. Age matched asymptomatic controls with no exposure risk for SARS-CoV-2 were defined by a discriminating set of six different bacterial species, thus suggesting a role for SARS-CoV-2 in altering the oropharyngeal microbiota or greater susceptibility to SARS-CoV-2 infection based on microbiome composition. While we found no differences in species abundance by traditional statistics between our COVID-19+ and COVID-19− patients, our SARS-CoV-2 negative patients were symptomatic and therefore could have been infected by other respiratory viruses, which may have altered their nasopharyngeal and oropharyngeal microbiomes. A study of the nasopharyngeal microbiomes of individuals with symptomatic upper respiratory tract infection either due to influenza or an or with unknown influenza status were similar, and show substantial changes compared to individuals who are asymptomatic (Kaul et al., 2020), this may have affected our ability to differentiate between COVID-19+ and COVID-19− individuals in this cohort. The identification of *P. salivae* and *V. infantium* by different sequencing methods and disparate geographic cohorts lends support to the importance of these two species in influencing COVID-19 symptoms. Interestingly, a pre-pandemic study on the effect of the oropharyngeal microbiome on susceptibility to symptomatic influenza infection found that an increased abundance of *P. salivae* was protective against symptomatic infection from a close household member (Tsang et al., 2020), again highlight a potential role for this organism in symptomatic respiratory virus infection. Our RFC modeling was able to provide a further layer of nuance in discriminating between symptomatic COVID-19+ patients who needed respiratory support and those who did not. If *Prevotella* and *Veillonella* are considered signatures of dysbiotic oral microbiota in SARS-CoV-2 respiratory illness, our analysis of encoded metabolic pathways provides additional insight into possible mechanisms by which these two species contribute to COVID-19 respiratory symptoms.

Our metabolic pathway analysis found that a decreased abundance of a LPS biosynthesis pathway, CMP-3-deoxy-D-*manno*-octulosonate, was the top predictor of requiring respiratory support. Additionally, another component of LPS biosynthesis (lipid IV A) was ranked 7th among the top 15 significant predictors of the need for respiratory support in acute COVID-19 (Figure 3D). CMP-3-deoxy-D-*manno*-octulosonate is a critical metabolite in LPS biosynthesis (Goldman et al., 1988), and lipid IV A is a precursor in the production of the lipid A core of LPS (Brozek and Raetz, 1990). Although it initially seems counter-intuitive that the abundance of pathways associated with the synthesis of LPS, known to generate substantial inflammation *via* activation of the innate immune system (Beutler and Poltorak, 2001), would be associated with less severe COVID-19 lung disease, we hypothesized that the detected LPS biosynthetic pathways were originating from species known to produce less inflammatory LPS. When we examined the contribution of bacterial genera to the CMP-3-deoxy-D-*manno*-octulosonate biosynthesis pathway, we observed that in patients who required respiratory support, less of the pathway originated from *Prevotella* and a larger portion originated from *Pseudomonas*, a known respiratory pathogen capable of producing highly inflammatory LPS (Goldberg and Pler, 1996). A possible explanation for these findings may be related to the natural history of COVID-19 lung disease. Sequencing-based analysis of broncho-alveolar lavage fluid from patients hospitalized with COVID-19 lung disease has shown the presence of oropharyngeal flora, which are hypothesized to enter the lungs by aspiration (Bao et al., 2020). The presence of organisms producing more inflammatory LPS in the oropharynx translocating to the lungs may potentiate inflammation during COVID-19 lung disease and lead to the need for respiratory support. Although our results can align with a model of *Prevotella* and *Veillonella* as “dysbiotic” species, our findings also support the hypothesis that a higher abundance of *Prevotella* and other species producing weakly immunogenic LPS corresponds to decreased abundance of more inflammatory LPS producing species. If aspiration and translocation occur during COVID-19, the presence of organisms that produce less inflammatory LPS may limit inflammation in the lungs of COVID-19 patients.

**Figure 4:**
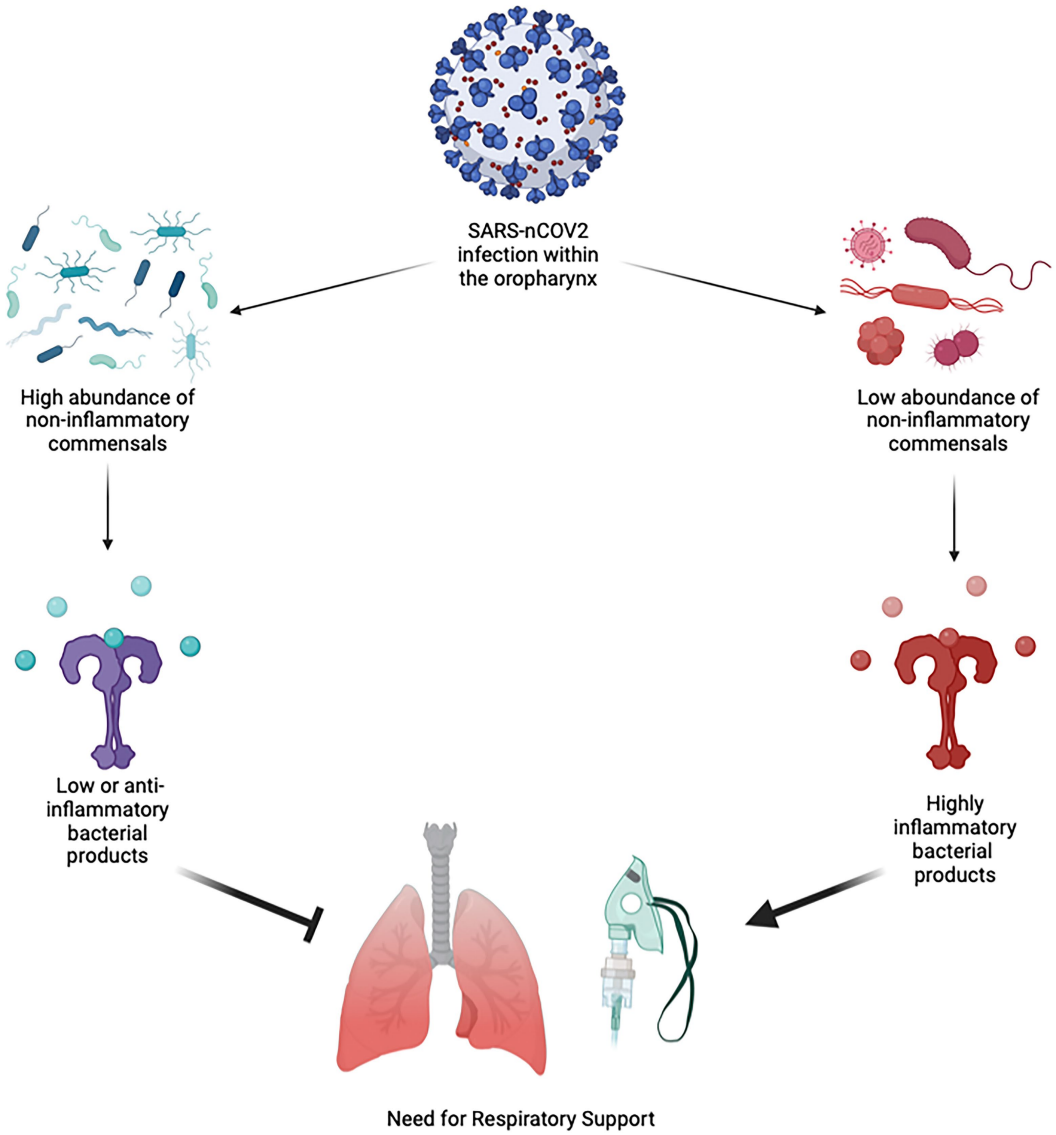
Conceptual Diagram of SARS-nCoV2 Interaction with the Oropharyngeal Microbiome. When an infection with SARS-nCoV2 begins within the oropharynx, it occurs within the environment of the microbiome. If the oropharyngeal microbiome has a high abundance of non-inflammatory or minimally inflammatory species that do not produce strongly inflammatory bacterial products, this will be protective against more severe lung disease and the need for respiratory support. If the oropharyngeal microbiome has a low abundance of non-inflammatory or minimally inflammatory species, then these produce more inflammatory bacterial products, leading to more severe lung disease and the need for respiratory support.

Our conceptual interpretation of the interplay between the oropharygeal microbiome and SARS-nCoV2 based on our results are shown in Figure 4. The predominant pattern that we observed within the oropharyngeal microbiome more generally was that a decrease in the abundance of several commensal organisms and an increase in abundance of bacterial products synthesis pathways is the primary predictor of the need for respiratory support in acute COVID-19. The finding that certain bacteria of the oropharyngeal microbiota are potentially protective against severe COVID-19 fits with observational data related to the treatment of COVID-19 patients with antibiotics. These studies suggest that treatment of COVID-19 with antibiotics does not reduce mortality and that secondary bacterial infection is uncommon (Chedid et al., 2021; Langford et al., 2021). Our findings run counter to the hypothesis that the oropharynx is primarily a source of opportunistic pathogens that gain access to the lungs during the course of COVID-19 (Bao et al., 2020).

If the predominant effect was that the presence of harmful or pathogenic bacteria in the oropharynx contributing to severe COVID-19 respiratory symptoms, one might expect treatment with antibiotics to be beneficial. Our findings are more consistent with the results of animal-model experiments with influenza, which suggest that treatment with antibiotics is potentially harmful due to their effect on beneficial commensal organisms. In mice challenged with influenza who had normal upper airway microbiota, macrophages activated genes associated with anti-viral activity such as interferon-gamma, while mice treated with antibiotics failed to activate these pathways and had more severe lung disease (Abt et al., 2012). In another study, antibiotic treatment prior to influenza challenge impaired dendritic cell priming and migration to draining lymph nodes that ultimately led to impaired development of T-cell mediated adaptive immunity (Ichinohe et al., 2011). In COVID-19, the oropharyngeal microbiome may play a similar role, aiding the development of an effective anti-viral response that limits severe disease manifestations. In this context, the microbiome was demonstrated to be critical to mounting an effective immune response to viral infection (Abt et al., 2012; Short et al., 2014). Thus, care should be exercised in analysis of “dysbiotic” profiles. On the one hand, there is evidence that host-microbiome interactions influence susceptibility to viral infection in the respiratory tract, while on the other hand, evidence also exists that suggests viral infections may cause dysbiosis. Development of disease may depend on a delicate interplay between these opposing forces. Future studies combining metagenomics, metabolomics, host biomarkers, host immune responses, and time series analysis would be of great value to understanding the complex interplay in the host-pathogen-microbiome axis.

### Strengths and limitations

Our strengths include our enrollment of patients within the Emergency Department during acute presentation of the disease, prospective clinical data collection, use of metagenomic sequencing, and use of two independent analysis techniques to verify our results. The majority of enrollment and collection of samples within the Emergency Department allowed us to sample the microbiomes of patients early in disease course before medical intervention. We did include some samples from our hospital’s COVID-19 treatment ward later in the course of the study. As such there were some samples that were collected from participants after they had received treatments such as antibiotics. However, treatment with antibiotics did not appear to affect the amount of microbial DNA that was obtained *via* these swabs (Supplementary Figure S1). Controlled studies of the effect of antibiotics on the oral microbiome suggest that subtle changes are seen 1 week after treatment antibiotic, but that differences peak at 1 month after exposure (Zaura et al., 2015). The longest possible period of time between antibiotic exposure and sample collection in this study was 4 days, and as such, we do not believe this represents a substantial confounder in this analysis.

We excluded any patients with self-reported symptoms longer than 14 days at time of collection to focus our analysis on the acute phase of the COVID-19. We chose 14 days as this is the point at which most COVID-19 patients will typically begin to recover from acute symptoms (Blair et al., 2021) and felt that beyond this time, respiratory decompensation could have been attributed to other causes. Our characterization of the oropharyngeal microbiome showed us features that can be predictive of disease course and potentially a target for therapeutics. In addition, the use of metagenomic sequencing for microbiome characterization enabled us to determine what bacterial metabolic pathways could potentially affect disease course as opposed to just genus-level information provided by 16S rRNA sequencing. Our interpretation of the results of the metabolic pathway abundances relies on the assumption that pathway abundance correlates with metabolite abundance, which has been previously shown to be reasonable in other systems (Mchardy et al., 2013), although direct detection of metabolite abundances would have strengthened our findings.

Weaknesses of this study include a single time-point in microbiome sampling from a single center and enrollment of a limited number of patients presenting with acute COVID-19 early in the disease course. Single time-point sampling does not allow for observation of how an individual’s oropharyngeal microbiota may change over the course of the disease. Although we enrolled 115 patients in the study, after focusing on the acute phase of COVID-19, only 50 COVID-19+ individuals with complete data were available for full analysis, which reduces statistical certainty. The reasons for incomplete data are multifactorial and include difficulties conducting clinical research during the early phases COVID-19 pandemic. We developed a method to limit research staff contact with patients to prevent the spread of COVID-19 by having nursing staff collect specimens during routine clinical care after verbal consent. Although we successfully protected our staff, this necessitated the need for follow up to collect information on symptoms and symptom duration, which is challenging among an Emergency Department population, and led to missing clinical data and later withdrawal of consent.

This study was conducted before widespread availability of vaccines against COVID-19, so although vaccination status is not a potential confounder in this study as no participants were vaccinated, how vaccination would change the interaction between the virus and the oropharyngeal microbiome is unknown. Vaccination likely affects the interaction of the host and virus in the oropharynx, and vaccination itself can affect the makeup of the oropharyngeal microbiota (Uehara et al., 2022). Studies of the oropharyngeal microbiome of COVID-19 among vaccinated patients will be required in the future to determine if the same interactions are seen.

## Conclusion

We demonstrate a relationship between disease manifestations of COVID-19 and the oropharyngeal microbiome. Specifically, the decreased abundance of some organisms, primarily *P. salivae*, is predictive of patients requiring respiratory support. We show that the presence of metabolic pathways for bacterial products such as LPS and mycolic acid are also predictive of not requiring respiratory support, implying that the presence of bacteria producing these products has a positive impact on disease course. Together, these findings suggest that the presence of beneficial commensal bacteria in the upper airway has the potential to prevent or mitigate pulmonary manifestations of COVID-19. We show that combining analysis of microbial abundances with metabolic pathways can provide deeper insight into microbiome “profiles” which may be predictive of certain clinical outcomes. Thus, our study underscores that the interaction between the oropharyngeal microbiome and respiratory viruses such as SARS-CoV-2 could potentially be harnessed for diagnostic and therapeutic purposes.

## Data availability statement

The datasets presented in this study can be found in online repositories. The names of the repository/repositories and accession number(s) can be found at: https://www.ncbi.nlm.nih.gov/, PRJNA735193.

## Ethics statement

The studies involving human participants were reviewed and approved by University of Massachusetts Institutional Review Board. The patients/participants provided their written informed consent to participate in this study.

## Author contributions

EB, JH, BM, and AM conceived and led the study. JH, EB, and CT supervised the conduct of the study and data collection. LC, M-CS, SM, CT, and PD managed the clinical data, including quality control. LC and M-CS handled the sample collection and storage. DW managed sample extraction and sequencing and performed metagenomic profiling. AZ and VB performed statistical analysis on the microbiome data and performed all ML modeling. EB, AZ, VB, and JH wrote the manuscript with input from all authors. All authors contributed to the article and approved the submitted version.

## Funding

This work was supported by the Society For Academic Emergency Medicine’s 2020 COVID-19 Research Grant, internal funding to support COVID-19 research early in the pandemic from the Dean of the University of Massachusetts Medical School and the National Institute of Health grant # 1RF1AG067483–01.

## Conflict of interest

The authors declare that the research was conducted in the absence of any commercial or financial relationships that could be construed as a potential conflict of interest.

## Publisher’s note

All claims expressed in this article are solely those of the authors and do not necessarily represent those of their affiliated organizations, or those of the publisher, the editors and the reviewers. Any product that may be evaluated in this article, or claim that may be made by its manufacturer, is not guaranteed or endorsed by the publisher.

## Footnotes

^1^ https://huttenhower.sph.harvard.edu/kneaddata/

^2^ https://github.com/an-bui/calecopal

## Supplementary material

SUPPLEMENTARY FIGURE S1 Proportion of metagenomic reads to host contamination. Shown is the ratio of metagenomic reads to host contamination reads in samples from COVID-19+ versus COVID-19- participants **(A)** and COVID-19+ participants requiring respiratory support versus those who did not **(B)**. Differences in the proportion of reads that were host contamination did not vary significantly between the groups (Mann-Whitney U-test p = 0.548 and p = 0.2383 respectively). We also stratified portion of metagenomic reads by participants who received antibiotics **(C)**, there was no significant difference in portion of metagenomic reads stratified by antibiotics recieved or number of antibiotics received (Mann-Whitney U-test for 1 antibiotic p = 0.5216, 2 antibiotics p= 0.6826, 3 antibiotics p = 0.8172).

SUPPLEMENTARY Table S1 Study population characteristics of COVID-19+ and COVID-19- cohorts.

SUPPLEMENTARY Table S2 Full statistics of Wilcoxon Rank Sum test comparing microbial abundance features between COVID-19+ and COVID-19- patients. Full results comparing species abundances between COVID-19- and COVID-19+ patients using the Wilcoxon Rank Sum test and Bonferroni correction for multiple comparisons.

SUPPLEMENTARY Table S3 Full statistics of Wilcoxon Rank Sum test comparing metabolic pathway features between COVID-19+ and COVID-19- patients. Full results comparing abundances of encoded metabolic synthesis pathways between COVID-19- and COVID-19+ patients using the Wilcoxon Rank Sum test and Bonferroni correction for multiple comparisons.

SUPPLEMENTARY Table S4 Full statistics of random forest classifier model to predict need for respiratory support based on microbial abundances. Full results from the 6 individual iterations of the RFC classifier model utilizing different datasets; bacterial abundances only, clinical covariates and Shannon diversity, clinical covariates and bacterial abundances, and combined bacterial abundance, clinical covariates and Shannon diversity. CC, Clinical Covariates; TP, True Positive; FP, False Positive; TN, True Negative; FN; False Negative.

SUPPLEMENTARY Table S5 Full statistics of random forest classifier model to predict need for respiratory support based on microbial abundances. Full results from the 6 individual iterations of the RFC classifier model utilizing metabolic pathway abundances; pathway abundance only and clinical covariates combined with pathway abundances. CC, Clinical Covariates; TP, True Positive; FP, False Positive; TN, True Negative; FN; False Negative.
