# Supplementary figures and images for: Oropharyngeal microbiome profiled at admission is predictive of the need for respiratory support among COVID-19 patients
Source: Front Microbiol. 2022 Sep 30;13:1009440. doi: 10.3389/fmicb.2022.1009440 (PMC9561819; doi:10.3389/fmicb.2022.1009440)

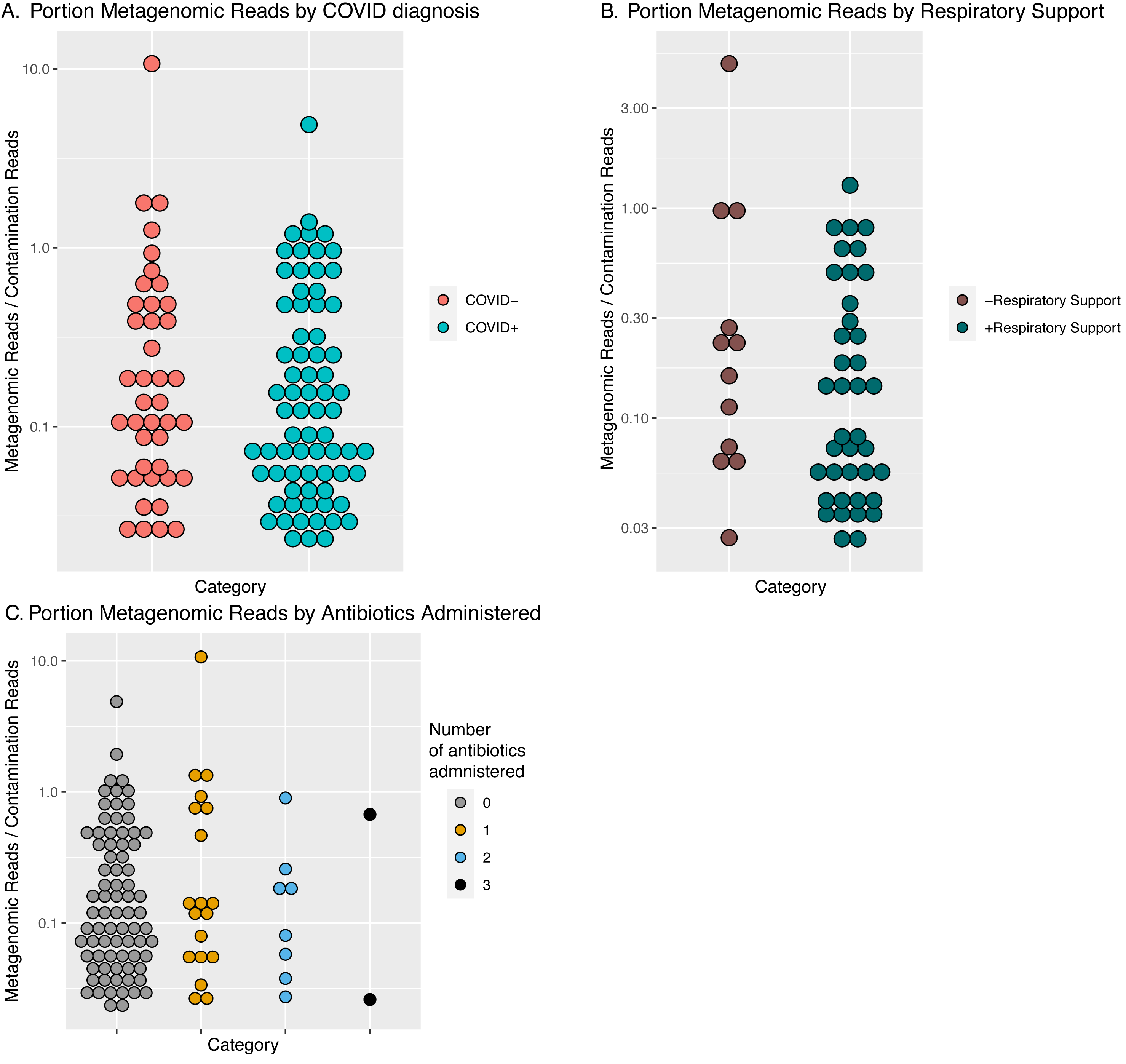

Supplement: SUPPLEMENTARY FIGURE S1 — Proportion of metagenomic reads to host contamination. Shown is the ratio of metagenomic reads to host contamination reads in samples from COVID-19+ versus COVID-19- participants (A) and COVID-19+ participants requiring respiratory support versus those who did not (B). Differences in the proportion of reads that were host contamination did not vary significantly between the groups (Mann-Whitney U-test p = 0.548 and p = 0.2383 respectively). We also stratified portion of metagenomic reads by participants who received antibiotics (C), there was no significant difference in portion of metagenomic reads stratified by antibiotics recieved or number of antibiotics received (Mann-Whitney U-test for 1 antibiotic p = 0.5216, 2 antibiotics p= 0.6826, 3 antibiotics p = 0.8172). [file Image_1.TIF]
