# Supplementary material for: Oropharyngeal microbiome profiled at admission is predictive of the need for respiratory support among COVID-19 patients
Source: Front Microbiol. 2022 Sep 30;13:1009440. doi: 10.3389/fmicb.2022.1009440 (PMC9561819; doi:10.3389/fmicb.2022.1009440)
Supplement: SUPPLEMENTARY Table S1 — Study population characteristics of COVID-19+ and COVID-19- cohorts. [file Table_1.DOCX]

**Table S1 COVID-19- versus COVID-19+ Study Population Characteristics**

| **Patient Characteristics** | **COVID-19-**  **(n = 41)** | **Total COVID-19+**  **(n =74)** | **P value** |
| --- | --- | --- | --- |
| Mean Age yrs (SD) | 65 (17.2) | 68.1 (16.2) | 0.39 |
| Female | 16 (39) | 38 (51.3) | 0.42 |
| Hispanic or Latino | 7 (17) | 19 (26) | 0.28 |
| White | 33 (80.5) | 49 (66.2) | 0.50 |
| Black | 1 (2.4) | 7 (9.5) | 0.13 |
| Asian | 1 (2.4) | 2 (2.7) | 0.28 |
| Other | 6 (14.6) | 16 (21.6) | 0.49 |
| CCI (sd) | 4.5 (2.8) | 4.39 (2.7) | 0.64 |
| Hypertension | 27 (65.8) | 47 (64.4) | 0.96 |
| Diabetes | 15 (36.6) | 22 (30.1) | 0.72 |
| Asthma | 5 (12.2) | 9 (12.3) | 0.52 |
| COPD | 7 (17) | 14 (19.2) | 0.68 |
| OSA | 7 (17) | 4 (5.5) | 0.023 |
| BMI | 28.8 (7.7) | 29.9 (7.4) | 0.40 |
| Active Smoker | 6 (14.6) | 1 (1.4) | <0.001 |
| Former smoker | 26 (63.4) | 31 (42.5) | 0.10 |
| ^a^ Data are presented as the number (%), unless otherwise specified.  *In patients who had a Do Not Intubate Order but suffered COVID-19 mortality, we considered them as having the highest requirement for respiratory support  COVID-19, Corona Virus Associated Infectious Disease-19;  CCI, Charlson Comorbidity Index; BMI, body mass index; COPD, Chronic Obstructive Pulmonary Disease; OSA, Obstructive Sleep Apnea  χ^2^ test was used to compare categoric variables and analysis of variance for continuous variables | | | |
