# Supplementary material for: Oropharyngeal microbiome profiled at admission is predictive of the need for respiratory support among COVID-19 patients
Source: Front Microbiol. 2022 Sep 30;13:1009440. doi: 10.3389/fmicb.2022.1009440 (PMC9561819; doi:10.3389/fmicb.2022.1009440)
Supplement: SUPPLEMENTARY Table S4 — Full statistics of random forest classifier model to predict need for respiratory support based on microbial abundances. Full results from the 6 individual iterations of the RFC classifier model utilizing different datasets; bacterial abundances only, clinical covariates and Shannon diversity, clinical covariates and bacterial abundances, and combined bacterial abundance, clinical covariates and Shannon diversity. CC, Clinical Covariates; TP, True Positive; FP, False Positive; TN, True Negative; FN; False Negative. [file Table_4.pdf]

| Model | Seed | TP | FP | TN | FN | Sensitivity | Specificity | Precision | Accuracy | F1 |
|---|---|---|---|---|---|---|---|---|---|---|
| Bacterial Abundance | 111 | 35 | 11 | 1 | 3 | 0.92 | 0.08 | 0.76 | 0.72 | 0.83 |
| Bacterial Abundance | 112 | 35 | 11 | 1 | 3 | 0.92 | 0.08 | 0.76 | 0.72 | 0.83 |
| Bacterial Abundance | 113 | 35 | 11 | 1 | 3 | 0.92 | 0.08 | 0.76 | 0.72 | 0.83 |
| Bacterial Abundance | 114 | 35 | 11 | 1 | 3 | 0.92 | 0.08 | 0.76 | 0.72 | 0.83 |
| Bacterial Abundance | 115 | 35 | 10 | 2 | 3 | 0.92 | 0.17 | 0.78 | 0.74 | 0.84 |
| Bacterial Abundance | 116 | 35 | 10 | 2 | 3 | 0.92 | 0.17 | 0.78 | 0.74 | 0.84 |
| CC + Shannon Diversity | 111 | 36 | 10 | 2 | 2 | 0.95 | 0.17 | 0.78 | 0.76 | 0.86 |
| CC + Shannon Diversity | 112 | 36 | 10 | 2 | 2 | 0.95 | 0.17 | 0.78 | 0.76 | 0.86 |
| CC + Shannon Diversity | 113 | 36 | 10 | 2 | 2 | 0.95 | 0.17 | 0.78 | 0.76 | 0.86 |
| CC + Shannon Diversity | 114 | 36 | 10 | 2 | 2 | 0.95 | 0.17 | 0.78 | 0.76 | 0.86 |
| CC + Shannon Diversity | 115 | 36 | 10 | 2 | 2 | 0.95 | 0.17 | 0.78 | 0.76 | 0.86 |
| CC + Shannon Diversity | 116 | 36 | 10 | 2 | 2 | 0.95 | 0.17 | 0.78 | 0.76 | 0.86 |
| CC + Bacterial Abundance | 111 | 35 | 9 | 3 | 3 | 0.92 | 0.25 | 0.80 | 0.76 | 0.85 |
| CC + Bacterial Abundance | 112 | 36 | 10 | 2 | 2 | 0.95 | 0.17 | 0.78 | 0.76 | 0.86 |
| CC + Bacterial Abundance | 113 | 36 | 11 | 1 | 2 | 0.95 | 0.08 | 0.77 | 0.74 | 0.85 |
| CC + Bacterial Abundance | 114 | 36 | 9 | 3 | 2 | 0.95 | 0.25 | 0.80 | 0.78 | 0.87 |
| CC + Bacterial Abundance | 115 | 36 | 10 | 2 | 2 | 0.95 | 0.17 | 0.78 | 0.76 | 0.86 |
| CC + Bacterial Abundance | 116 | 35 | 10 | 2 | 3 | 0.92 | 0.17 | 0.78 | 0.74 | 0.84 |
| CC + Bacterial Abund. + Shannon Div. | 111 | 36 | 10 | 2 | 2 | 0.95 | 0.17 | 0.78 | 0.76 | 0.86 |
| CC + Bacterial Abund. + Shannon Div. | 112 | 36 | 9 | 3 | 2 | 0.95 | 0.25 | 0.80 | 0.78 | 0.87 |
| CC + Bacterial Abund. + Shannon Div. | 113 | 36 | 9 | 3 | 2 | 0.95 | 0.25 | 0.80 | 0.78 | 0.87 |
| CC + Bacterial Abund. + Shannon Div. | 114 | 36 | 10 | 2 | 2 | 0.95 | 0.17 | 0.78 | 0.76 | 0.86 |
| CC + Bacterial Abund. + Shannon Div. | 115 | 35 | 9 | 3 | 3 | 0.92 | 0.25 | 0.80 | 0.76 | 0.85 |
| CC + Bacterial Abund. + Shannon Div. | 116 | 35 | 10 | 2 | 3 | 0.92 | 0.17 | 0.78 | 0.74 | 0.84 |
