# Supplementary material for: Oropharyngeal microbiome profiled at admission is predictive of the need for respiratory support among COVID-19 patients
Source: Front Microbiol. 2022 Sep 30;13:1009440. doi: 10.3389/fmicb.2022.1009440 (PMC9561819; doi:10.3389/fmicb.2022.1009440)
Supplement: SUPPLEMENTARY Table S5 — Full statistics of random forest classifier model to predict need for respiratory support based on microbial abundances. Full results from the 6 individual iterations of the RFC classifier model utilizing metabolic pathway abundances; pathway abundance only and clinical covariates combined with pathway abundances. CC, Clinical Covariates; TP, True Positive; FP, False Positive; TN, True Negative; FN; False Negative. [file Table_5.pdf]

Model	seed	TP	FP	TN	FN	sensitivity	specificity	precision	accuracy	F1
Pathway Abundance	111.00	33.00	11.00	1.00	5.00	0.87	0.08	0.75	0.68	0.80
Pathway Abundance	112.00	34.00	12.00	0.00	4.00	0.89	0.00	0.74	0.68	0.81
Pathway Abundance	113.00	34.00	12.00	0.00	4.00	0.89	0.00	0.74	0.68	0.81
Pathway Abundance	114.00	33.00	11.00	1.00	5.00	0.87	0.08	0.75	0.68	0.80
Pathway Abundance	115.00	32.00	12.00	0.00	6.00	0.84	0.00	0.73	0.64	0.78
Pathway Abundance	116.00	33.00	11.00	1.00	5.00	0.87	0.08	0.75	0.68	0.80
Pathway Abund. + CC	111.00	34.00	11.00	1.00	4.00	0.89	0.08	0.76	0.70	0.82
Pathway Abund. + CC	112.00	35.00	10.00	2.00	3.00	0.92	0.17	0.78	0.74	0.84
Pathway Abund. + CC	113.00	34.00	11.00	1.00	4.00	0.89	0.08	0.76	0.70	0.82
Pathway Abund. + CC	114.00	34.00	10.00	2.00	4.00	0.89	0.17	0.77	0.72	0.83
Pathway Abund. + CC	115.00	34.00	11.00	1.00	4.00	0.89	0.08	0.76	0.70	0.82
Pathway Abund. + CC	116.00	34.00	10.00	2.00	4.00	0.89	0.17	0.77	0.72	0.83
